# Discrimination experiences and their associations with sociodemographic factors, health and quality of life—a latent class analysis

**DOI:** 10.1186/s12955-026-02502-2

**Published:** 2026-02-19

**Authors:** Jan Georg Friesinger, Dina von Heimburg, Ottar Ness, Siri Håvås Haugland, John-Kåre Vederhus

**Affiliations:** 1 Department of Psychosocial Health, University of Agder, Grimstad, Norway; 2 Department of Education and Lifelong Learning, Norwegian University of Science and Technology, Trondheim, Norway; 3 Addiction Unit, Sørlandet Hospital, Kristiansand, Norway

**Keywords:** Discrimination, Quality of life, Health, Survey, Norway

## Abstract

**Background:**

Discrimination has adverse effects on people’s health and quality of life (QoL). This study aimed to identify distinct intersecting patterns of discrimination experiences, examine their associations with social categories and health factors, and assess the impact of perceived discrimination (PD) on health and QoL outcomes beyond specific self-reported reasons for PD.

**Methods:**

We utilized data from the Norwegian Counties Public Health Survey (NCPHS) conducted in Agder County in 2023 and employed latent class analysis (LCA) to explore how patterns of discrimination reasons cluster. The selected classes were then further examined to determine how they differed by comparing each class to the reference class with no PD. Lastly, we assessed the estimated marginal means of each class on health and overall QoL using ANCOVA.

**Results:**

The study identified six classes of PD: Massive PD, Gender/Age PD, No PD, Function/Illness PD, Ethnicity/Skin PD, and Political PD. ANCOVA analyses revealed significant differences across self-rated health, mental distress, and QoL. Notably, the Massive PD, Gender/Age PD, and Function/Illness PD groups reported significantly poorer self-rated health and QoL compared to the No PD group. All PD classes scored significantly higher in mental distress than the No PD group, with the Massive PD class exceeding the clinical cut-off, indicating elevated psychological distress.

**Conclusions:**

Our findings reveal persistent health and QoL disparities between individuals experiencing PD and those who do not, despite a robust welfare system. Service providers must consider the interplay of factors such as age, gender, income, and health conditions with PD to ensure fair service delivery.

**Supplementary Information:**

The online version contains supplementary material available at 10.1186/s12955-026-02502-2.

## Introduction

Despite legal protection, discrimination is a widespread phenomenon which is understood as the unfair treatment of a person or group for reasons such as age, gender, ethnicity, health, sexuality, socioeconomic status (SES), religious or political affiliations [1]. Discrimination is conceptually related to stigma and is built upon affective, cognitive, and social components [2]. It involves stereotypes, which are negative emotions toward a person or group, and prejudice, which consists of negative beliefs about the person or group. These components lead to discriminatory actions or behaviors against them and operate on interpersonal and systemic levels [3, 4]. For example, ageism, sexism, and racism refer to stereotypes, prejudice, and discrimination against people based on their chronological age, gender, and ethnic background, respectively [5–7]. A young woman might face ageism when her professional capabilities are questioned due to her youth, sexism when she’s subject to gender-biased expectations, and racism if assumptions are made about her based on her ethnic background or skin color. Outcomes of such perceived discrimination (PD) range from psychological distress (e.g., anxiety, depression, lowered self‑esteem) and chronic stress–related physical problems to reduced educational and employment opportunities, lower income, social isolation, and decreased quality of life (QoL) [8].

Discrimination reasons can intersect, such as shown by the example of the young woman, and there is an interest in identifying distinct patterns of discrimination, which is one of the aims of this study. Across societies, epidemiologists have explored the adverse effects of discrimination on people’s health and found that it contributes to health inequities [8–11]. Particularly, racism is a severe form of discrimination [12–15]. When assessing discrimination through an individual’s perception, previous research has shown that PD negatively impacts both physical and mental health [10, 16, 17], with effects varying across population subgroups [18]. Likewise, people’s overall QoL is also significantly reduced [19, 20].

Moreover, scholars indicate that experiencing multiple forms or specific combinations of discrimination together may worsen the negative impact on people’s health and wellbeing [21–23]. Intersectionality, as a theoretical framework, highlights the importance of considering multiple overlapping sources of disadvantage when explaining health disparities [24–26]. When social identities (e.g. skin color, gender, SES) overlap, they can interact to influence health and QoL in ways that exceed a simple cumulative effect of each factor [27–29]. For this reason, an intersectional perspective is valuable for understanding health equity, as it highlights how intersecting identities (such as gender, ethnicity, SES, disability and migration background) produce distinct patterns of advantages and disadvantages [30]. For example, a recent Norwegian rapport highlighted that several groups scored low in subjective wellbeing in cases of intersections between low SES, unemployment, disabilities, mental illness, LGBTQ+, single household or exposure to discrimination/social exclusion [31]. Lewis et al. identified a knowledge gap concerning our understanding of how intersectionality forms the consequences of discrimination [32].

Critical voices on intersectionality highlight theoretical and methodological challenges, yet they also acknowledge the positive contributions of an intersectional approach to public health and QoL research [8, 24, 33, 34]. An issue, for example, is the lack of clear guidelines for quantitative studies on how to operationalize intersectionality [27]. In a previous study, we found that cumulative exposure to multiple reasons for discrimination was associated with poorer self-rated health and/or mental distress [16]. However, we did not examine how patterns of discrimination cluster to reflect intersecting reasons for discrimination, a focus of the current article, where we applied latent class analysis (LCA), along with exploring their associations with QoL. As such, our article seeks to contribute to QoL research inspired by an intersectional perspective.

### Objectives

The objectives of the present study were the identification of latent classes of perceived discrimination (PD), their clustering with social and health characteristics, and their associations with health and quality of life [35].

We hypothesized that latent class analysis (LCA) will identify distinct classes of PD characterized as intersection of PD categories rather than a single homogeneous profile of discrimination experiences. We further hypothesized that membership of the identified latent classes will be clearly associated with background social categories and health-related characteristics — specifically with age, gender, education, financial difficulties, living alone, immigrant background, long-term health problems, and functional impairments. In line with previous research, we expected that elevated levels of PD, as represented by the latent classes, would be associated with poorer health (i.e., mental health and self-rated overall health) and lower QoL compared with the “No PD” class.

## Methods

The Norwegian Counties Public Health Survey (NCPHS) collects data from adults (18+) across counties to inform municipal and county-level public health planning [36]. The survey was conducted in repeated waves and covers health, demographic, and socioeconomic indicators. This study used data from the NCPHS in collaboration with the Norwegian Institute of Public Health (NIPH) and the Agder County Council [37]. A random sample of 57,891 residents was drawn from the Population Registry, with contact details obtained from the Agency for Public Management and eGovernment. Data was collected from September 20 to October 23, 2023, with 18,517 residents participating (32.0% response rate). Once the data were collected and stored, they were encrypted and transferred to a secure server at the NIPH for further processing and analysis. Following approval for ethical and data privacy protection, the NIPH made the data accessible to a project group at the University of Agder.

### Measures

The study included background variables: age, sex, education (high = ≥ bachelor’s, low = < bachelor’s), living situation (alone/not), and immigrant background (respondent or parent born outside Norway). Financial situation was measured on a six-point scale of managing daily expenses and dichotomized into financial difficulties (somewhat difficult or worse) and no difficulties. Health status was assessed by self-reported long-term illness and functional impairments (yes/no). Those who reported being very much bothered were categorized as having substantial health problems or impairments.

#### Perceived discrimination (PD)

PD was assessed by asking participants whether, in the past 12 months, they had been treated worse than others for any of the following reasons: age, gender, illness (health problems, illness, injury), function (disability), ethnic background, skin color, religion/beliefs, political views, sexual identity or other reason. Responses were recorded as yes or no. ‘Other reason’ was excluded from the LCA because of its ambiguity and difficulty in classification.

#### Health outcomes and QoL

*Self-rated health* was assessed with a single question [38], with responses on a 5-point scale ranging from “very bad” (1) to “very good” (5) [39]. *Mental distress* was measured using the 5-item version of the Hopkins Symptom Checklist. The respondents rated how much they had felt sad or depressed, hopeless about the future, tense or anxious, constantly fearful, or worried in the past week on a 4-point scale from “not bothered” to “extremely bothered”. Scores were averaged, with a mean of ≥ 2.0 indicating clinically significant mental distress [40]. *QoL* was measured using the Essential QoL-3 (EQoL-3) scale, which assesses perceived satisfaction with life, happiness, and meaningfulness. The participants rated each item on a scale of 0 to 10, with higher scores indicating greater satisfaction, happiness, and perceived meaningfulness. Scores were averaged to provide an overall QoL score [41].

### Statistics

We used latent class analysis (LCA) to identify distinct intersecting patterns of discrimination experiences [42, 43]. As a person-centered approach, LCA examines similarities and differences among individuals regarding how their experiences of discrimination relate to one another, thereby categorizing individuals into ‘classes’ on the basis of these shared experiences [44, 45]. This analysis reveals whether certain types of discrimination are commonly clustered. The experience of the previously described reasons for discrimination in the last 12 months (categorical yes–no responses) was used as indicators of class membership. With nine indicators, we considered models with up to eight classes. To determine the optimal number of classes, we used the likelihood ratio chi-square (LRX^2^) as an absolute fit index, with non-significant values indicating good fit [46]. We also compared models using the Bayesian Information Criterion (BIC), where lower values indicate a better model. Classification accuracy was assessed using entropy, a summary measure of classification quality, where higher values indicate better accuracy. A value greater than 0.8 is considered indicative of high classification quality [47]. Additionally, we report the average latent class probabilities for each class, that is, the average probability of respondents being assigned to their most likely latent class—to further assess classification accuracy. An acceptable classification is when the probability of correct class membership assignment is ≥ 0.7 [47]. The final model, which demonstrated favorable fit indices and yielded substantively meaningful and interpretable classes, was then selected. We then transferred the data to SPSS ver. 30 for further analysis, examining how the classes differed in sociodemographic and health factors by comparing each to the reference class with no PD. Chi-square tests were used for categorical variables, and Student’s t-tests were used for continuous variables. Finally, we assessed the estimated marginal means of each class on subjective health and overall QoL using ANCOVA (Analysis of covariance), adjusting for sociodemographic factors and health variables, including long-term illnesses/health problems and functional impairment. We used R² to report the proportion of variance explained by the models. These analyses aimed to identify the influence of sociodemographic factors, health problems, and functional impairments on perceived health and QoL beyond the impact of the underlying characteristics associated with different types of PDs.

## Results

### Descriptive statistics

In analyzing the data for this study, 110 respondents with missing data for all the PD variables were excluded from the results presented below. Consequently, a total of 18,407 respondents were included in the analysis. The participants had a mean age of 51.6 (± 16.5) years; 56% were female, and 57% had an education below a bachelor’s degree (Table 1). Furthermore, 1 in 5 participants lived alone, 1 in 4 faced financial difficulties, and one in six had an immigrant background. Additionally, 15.7% were very much bothered by long-term illnesses or health problems, whereas 7.5% reported significant issues with functional impairments or problems related to an injury (Table 1).

**Table 1:** Sample characteristics of the study participants (*N* = 18,407)

	*N* (%)
Age (years, means (±))	51.6 (± 16.5)
Sex (female)	10,269 (55.8)
Educational level (*N* = 18,301)	
Less than Bachelor’s degree	10,352 (56.6)
Bachelor’s degree or higher	7,949 (43.4)
Living alone (*N* = 18,407)	3,485 (18.9)
Immigrant background (*N* = 18,331)	2,994 (16.3)
Financial difficulties (*N* = 17,995)	4,706 (26.2)
Very much bothered by long-term illnesses or health problems (*N* = 18,204)	2,865 (15.7)
Very much bothered by functional impairment or problems due to injury (*N* = 18,206)	1,363 (7.5)

### Latent class analysis

Table 2 presents fit indices for models up to 8 classes. Entropy was high across the models, reflecting low classification uncertainty. The LRΧ² test indicated that models with six to eight classes had an acceptable fit (non-significant values). The BIC suggested that a six-class model provided the best fit. This model included one boundary parameter estimate—values at the edge of the theoretically admissible parameter space. While this is not optimal, only one such estimate is acceptable. The seven- and eight-class models, on the other hand, had more boundary parameter estimates, which is a common issue in models with a large number of classes [46]. These boundary estimates overlapped between classes, complicating interpretation and suggesting over-extraction of classes. Therefore, considering both the BIC and the improved interpretability, the six-class model was deemed the most parsimonious and was selected as the final model.

**Table 2:** Fit indices for the latent class models (*N* = 18.407)^a^

	LL^a^	X^2^_LRT (df),_*p*-value	Entrophy	BIC
2 Class	-19,693	1354 (df 395), *p* < 0.001	0.85	39,574
3 Class	-19,194	836 (df 398), *p* < 0.001	0.92	38,674
4 Class	-18,982	654 (df 396), *p* < 0.001	0.87	38,347
5 Class	-18,857	496 (df 398), *p* < 0.001	0.87	38,197
**6 Class**	**-18,780**	**343 (df 385)**, ***p* = 0.938**	**0.89**	**38,140**
7 Class	-18,737	255 (df 373), *p* = 1.00	0.91	38,153
8 Class	-18,717	233 (df 369), *p* = 1.00	0.88	38,209

Note: ^a^*N* = 110 was excluded from the LCA analysis because all perceived discrimination variables were missing. LL = log likelihood values. X^2^_LRT (df)_ = Chi-square Likelihood Ratio Test (degrees of freedom). BIC = Bayesian Information Criterion

**Table 3:**
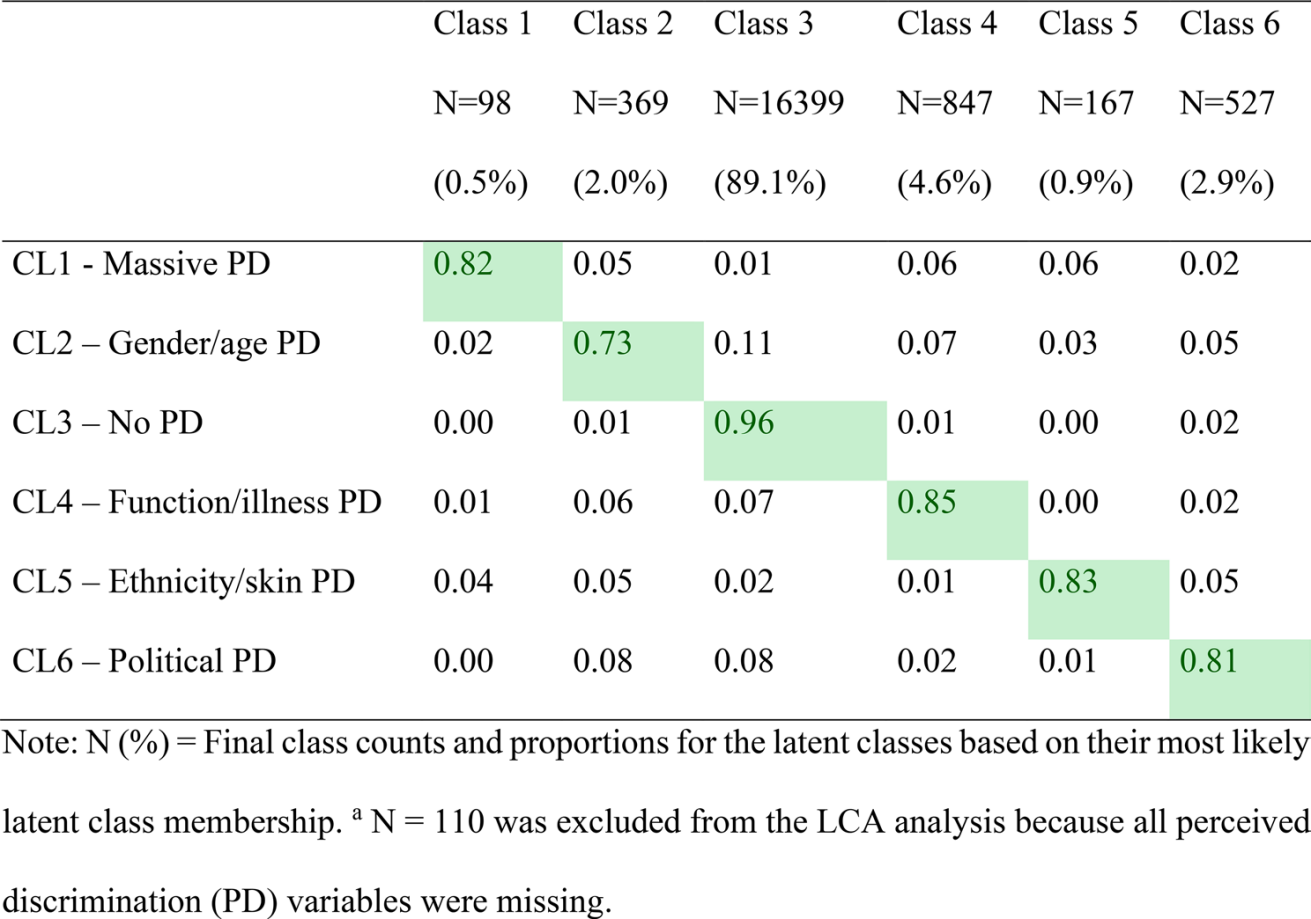
Average latent class probabilities for the most likely latent class membership (row) by latent class (column) (*N* = 18.407)^a^

In the six-class model, the average latent class probabilities for the most likely latent class membership were generally high (entropy = 0.89), with most classes showing strong latent class probabilities that largely excluded membership in other classes with all probabilities exceeding the recommended threshold of 0.7 (Table 3).

**Fig. 1:**
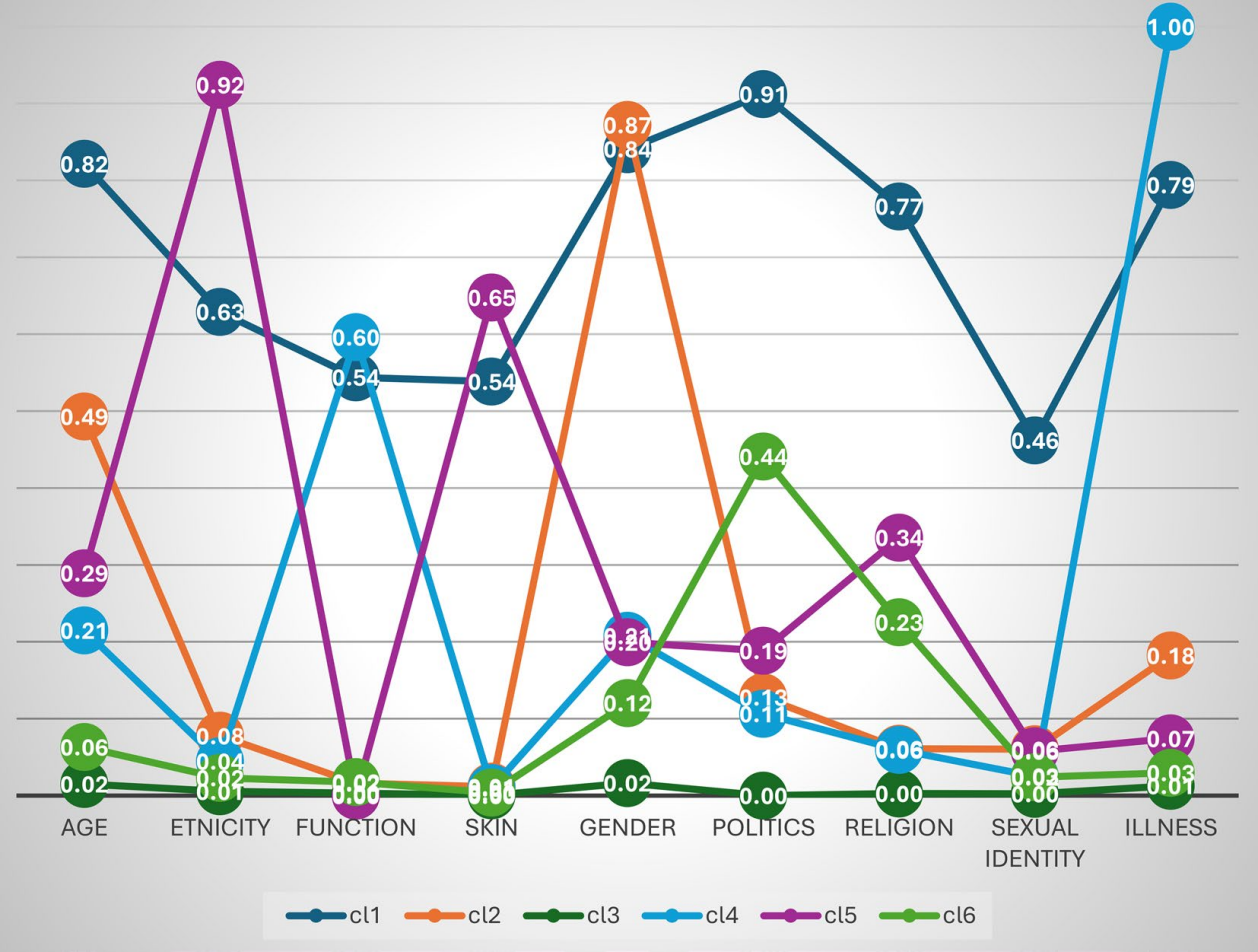
Latent Class Analysis (LCA) profiles based on reasons for perceived discrimination (PD)

Figure 1 illustrates the latent class profiles for this model, with class labels reflecting the clustering of experiences. Class 1, the smallest group, exhibited a moderate to high probability of experiencing PD across all categories and was labeled “Massive PD”. Class 2, which presented a high probability of gender-based PD and a moderate probability of age-related PD, led to the label “Gender/Age PD”. Class 3, the largest group, comprising 89.1% of the sample, had minimal PD experiences and was labeled “No PD”. Consequently, about 1 in 10 individuals (10.9%) were classified into one of the PD classes. Class 4, the largest of the PD classes, had a high probability of PD related to functional limitations and health issues, earning it the label “Function/Illness PD”. Class 5 showed high probabilities of ethnicity- and skin-related PD, along with a modest probability of religion-based PD, and was labeled “Ethnicity/Skin PD”. Finally, Class 6 presented moderate probabilities of PD on the basis of political views, with a modest probability of religion-based PD and low probabilities elsewhere, leading to the label “Political PD”.

### Characteristics of the latent classes

**Table 4:**
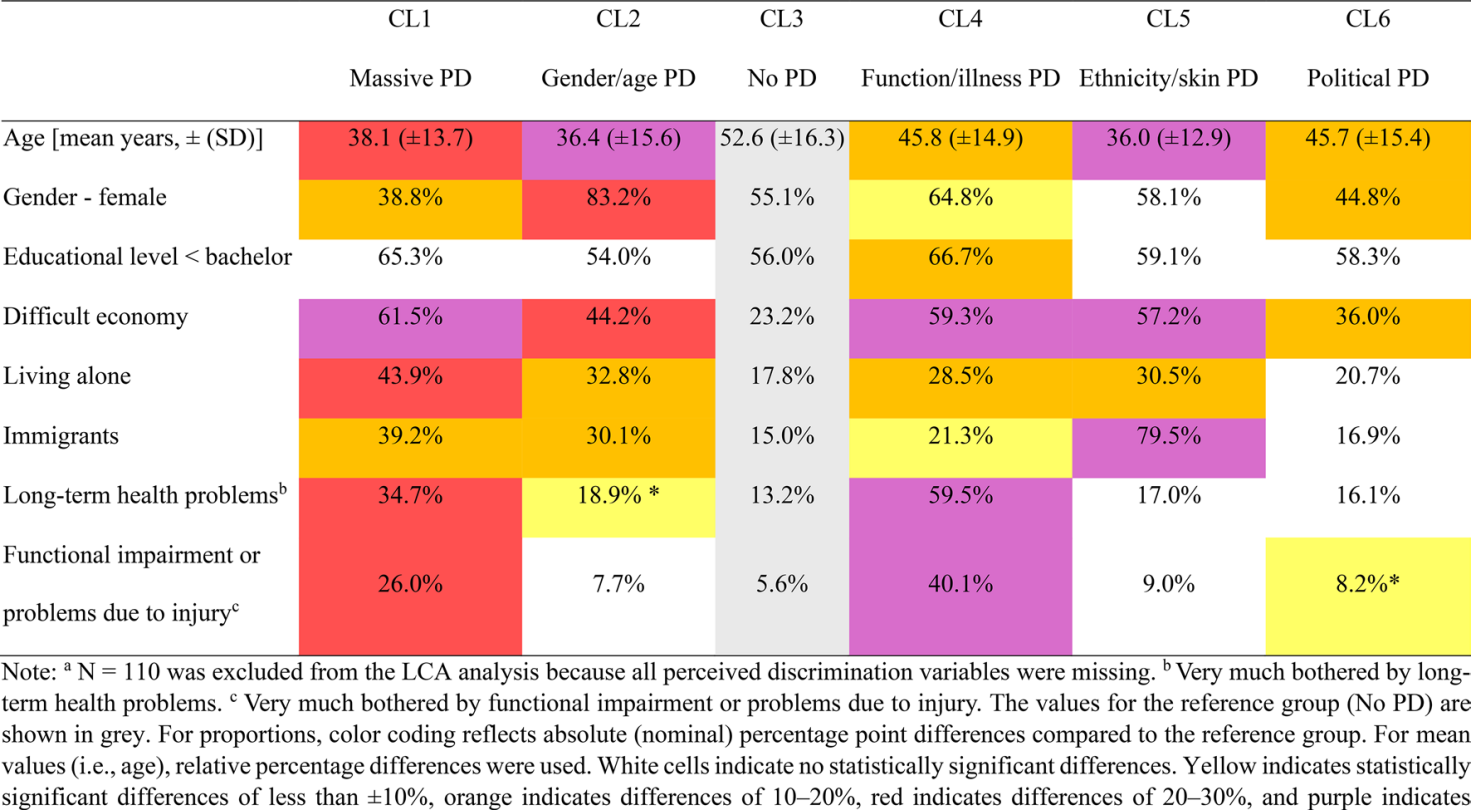
Sociodemographic, health, and functional characteristics of the latent classes, highlighting deviations from the reference group with no perceived discrimination (No PD) (*N* = 18.407)^a^

To examine how the classes were characterized in terms of sociodemographic and health factors, we compared each to the reference class with no PD (Table 4). Focusing on the largest deviations from the reference class, the key characteristics were as follows: CL1 (Massive PD) was characterized by a greater proportion of younger respondents and respondents with financial difficulties, living alone, and reporting long-term health problems and functional impairments. CL2 (Gender/age PD) had a substantially younger age profile and a much greater proportion of females. CL4 (Function/illness PD) had markedly higher proportions of individuals with long-term health problems and functional impairments, as well as a much greater proportion experiencing financial difficulties. CL5 (Ethnicity/skin PD) was much younger than the reference class and included a much greater proportion of immigrants and more individuals with financial difficulties. CL6 (Political PD) did not show extreme deviations but had a slightly younger age profile, a lower proportion of females, and a somewhat higher proportion experiencing financial difficulties. Educational level did not differ significantly from the reference class in any class, except for a slightly higher proportion with lower education in CL4.

### Analysis of covariance

**Fig. 2:**
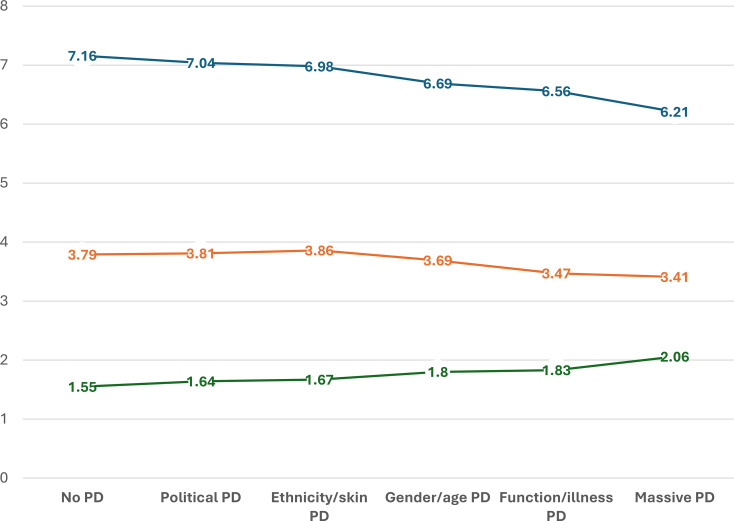
Health and quality of life (QoL) outcomes across the latent classes. Note: Estimated mean values from ANCOVA analyses for the different perceived discrimination (PD) classes, adjusted for age, gender, living alone, educational level, financial difficulty, long-term illness/health problems, and functional impairment. The blue line represents the QoL score (EQoL-3), the orange line represents self-rated health, and the green line represents mental distress

Figure 2 presents health and QoL outcomes across the latent classes. Based on preliminary unadjusted analyses, we ordered the classes from best to worst outcomes (see supplemental File). ANCOVA results (adjusted analyses) revealed highly significant trends across all three outcome measures: self-rated health (F = 546, df = 16, *p* < 0.001, adjusted R^2^ = 0.33), mental distress (F = 634, df = 16, *p* < 0.001, adjusted R^2^ = 0.37), and QoL (F = 629, df = 16, *p* < 0.001, adjusted R^2^ = 0.36). For both self-rated health and EQoL-3, the three highest-ordered PD classes differed significantly from the reference group with No PD, with most comparisons reaching *p* < 0.001 and all below 0.013. In contrast, differences for the Political PD and Ethnicity/Skin PD classes were not statistically significant. For mental distress, all PD classes showed significant differences compared to the No PD class, with most *p* values below 0.001. In terms of self-rated health, the two highest-ordered classes, Function/Illness PD and Massive PD, scored approximately 0.3 points lower on the 1–5 scale compared to the No PD group. A similar pattern was observed for EQoL-3, with a decrease of 0.6 to nearly 1 point on the 0–10 scale from the No PD class to the two highest-ordered classes. This indicates a marked reduction in QoL. Mental distress followed a parallel trend in the opposite direction: the Massive PD group had a mean score above the clinical cut-off (> 2.0), indicating elevated psychological distress, whereas the Function/Illness PD group had scores approaching the clinical threshold (Fig. 2).

## Discussion

Our LCA identified some intersecting patterns of discrimination experiences, including a gender/age class, a function/illness class, and a ‘massive PD’ class characterized by reports of PD for multiple reasons. The function/illness cluster suggests a shared underlying dimension—such as stigma linked to perceived health vulnerability or deviation from normative functioning. Similarly, the ethnicity/skin color class was associated primarily with immigrant background, indicating that these experiences may be shaped by a common process of ethno-cultural classification rather than other separable categories. Thus, as LCA yields distinguishable classes, it was primarily the gender/age PD class and the massive PD class that could be interpreted as reflecting clustered patterns of discrimination.

When the results of the LCA were used in subsequent analyses, the classes presented distinct sociodemographic and health profiles, suggesting complex relationships between the PD classes and underlying social categories and background factors. Compared to the ‘No PD’ class, the sociodemographic composition in the other classes suggests underlying intersectional factors that may influence experiences of PD. This was particularly pronounced in the Massive PD class, which was characterized by a higher proportion of males, greater financial difficulties, a larger number of immigrants, and more individuals living alone. There were consistently high proportions reporting financial difficulties across most classes, particularly Massive PD, Function/Illness PD, and Ethnicity/Skin PD.

The National Quality of Life Strategy for Norway (2025–2030) highlights that people with low education, economic difficulties, unemployment, or disability consistently report lower QoL than the rest of the population does [48]. The multiple classes of PD examined in this study did not include poverty or low SES as a stand-alone class. However, Goldblatt et al. [31] emphasized that poverty is not only a material disadvantage but also tied to psychosocial harm—loss of control, reduced dignity, and stigma. Shame is often the emotional expression of this denied significance, where various forms of discrimination exacerbate these harms, particularly for those already struggling economically [31, 49]. These experiences are not adequately explained by sociodemographic factors alone; they are relational and systemic.

With respect to living alone, a similar pattern was observed, indicating that the absence of close companionship may increase vulnerability to discrimination. Related research with an extended focus on loneliness has shown that PD may influence health mediated by loneliness [50], a factor we did not directly account for in our study, as we only measured living alone, which is not synonymous with loneliness but may serve as a proxy. Females were much more likely to experience PD, which is consistent with previous research [51].

As expected, the Function/illness class included a much greater proportion of individuals with long-term illness, health problems, and functional impairments. This class was also characterized by financial difficulties, which is not surprising given the impact of health limitations and functional impairments on earning capacity and employment opportunities, particularly in later life [43]. Our findings further suggest that experiences of discrimination related to ethnicity and skin color are, as expected, closely and inherently linked to immigrant background. This group also faces greater risks of financial hardship and living alone, factors that may compound their vulnerabilities across social, economic, and ultimately health outcomes [13, 21, 52]. Similarly, a recent Norwegian study on minority stress showed that discrimination and stigma contribute to chronic stress, reduced mental health, and feelings of shame and invisibility. Many minorities report living with a constant burden of monitoring how others perceive them, which erodes QoL [53].

Women made up the majority of the Gender/Age class, indicating that PD related to gender/age is closely linked to gendered experiences [54]. Given that a younger age profile may be more likely to report discrimination, this could be seen as part of ‘youngism’, which is a form of ageism directed against younger people [55]. The Political PD class appeared less clearly defined, both in the LCA output and in its moderate deviations from the reference group—characterized only by a somewhat younger age profile, a greater proportion of men, and a somewhat greater proportion experiencing financial difficulties. Although somewhat speculative, these sociodemographic attributes may be associated with holding controversial political views, which in turn could be linked to experiences of discrimination [56].

Analyzing the impact of PD on subjective health and QoL, we adjusted for sociodemographic characteristics using ANCOVA, to isolate the net effect of PD. These background factors clearly influenced subjective health on their own, as shown by differences between unadjusted (see supplemental file) and adjusted analyses. The unadjusted results revealed greater variability in self-rated health and QoL across classes; however, after adjustment, substantial deviations from the reference class were primarily observed in the Function/Illness and Massive PD classes. In contrast, PD related to the other classes had a modest influence on subjective health and QoL, suggesting discrimination occurs across various domains.

However, a closer look at the relationships among PD, psychological distress (the subjective, transdiagnostic experience of mental discomfort) [57], health‑related QoL, and psychological well‑being (measured as positive affect and life satisfaction) [58] shows that age is an important moderator with clear clinical implications. Our findings suggest that younger age interacts with other characteristics (e.g., gender and ethnicity). For instance, a young woman from a marginalized background may experience compounded barriers to care and poorer psychosocial outcomes beyond those associated with age alone. A concrete implication is to link health-equity strategies to the implementation of Norway’s national action plan against racism and discrimination (2024–2027) [59], prioritizing measures in workplaces and local communities where many young adults spend much of their time. Moreover, staff in the health, social‑care, and education sectors should receive enhanced diversity training and be able to provide advice and guidance to those exposed to racism and discrimination.

According to our results, service providers should be aware that multiple forms of PD exist, even within a society often regarded as egalitarian [16, 17], with PD related to function and illness affecting the largest group. This highlights the need for intersectional approaches in health and social policy such as services that lessen the burden on younger people (e.g., targeted support for young adult carers and families affected by illness/disability) [31, 60]. Universal services and poverty reduction remain vital but insufficient; support must be proportionate to need and tailored to those facing intersecting disadvantages [61]. Public services and structural interventions should move beyond single-axis approaches. Effective interventions should integrate economic support with anti-discrimination measures and inclusive policymaking, ensuring dignity, voice, and equity. Addressing these dimensions together is essential for health equity and for enabling everyone to feel that they truly matter [49, 62]. Such multifaceted and systemic approaches to social change are essential to strengthen health equity and reduce social gradients in health [63, 64].

### Methodological considerations

Among the limitations of the study are issues related to the measurement of self-reported discrimination [32]. It has been questioned whether perceived discrimination accurately reflects actual experiences, as responses may be subject to minimization or exaggeration biases [65]. Our PD questions used a dichotomous (yes/no) response format, resulting in categorical data without information about the severity of experiences. Additionally, the study relied on retrospective self-reports. Our results are constrained by the study’s cross‑sectional design, which prevents assessment of temporal relationships and changes in exposure (of discrimination) or outcome over time and therefore limits causal inference. We also note that when assessing outcomes, it is not possible to statistically control for all potential confounding factors, which introduces a degree of uncertainty [28]. Health‑related non‑response bias could affect the results but appears to play a minor role, according to a recent study of the NCPHS [66]. These limitations should be considered when our findings are interpreted. Given that background factors such as financial hardship and living alone were associated with several PD classes, future studies might consider including items on discrimination related to poverty or social isolation. Nonetheless, the study’s strengths include a large, randomly drawn sample from a county population and the inclusion of a broad range of relevant PD experiences.

## Conclusions

Overall, our findings indicate that despite a robust welfare system, significant disparities in health and QoL persist between those who experience PD and those who do not. Factors such as age, gender, income, living situation, migration background, long-term health conditions, and functional impairments appear to combine in distinct patterns with PD, likely influencing individuals’ experiences and contributing, along with PD, to their health outcomes. In sum, our findings show that discrimination, particularly when experienced together and intersecting with other minority identities and poverty, poses serious risks to health and wellbeing. Service providers and policymakers should recognize that multiple forms of PD often co-occur and promote inclusive community development and equitable service delivery, tailored to diverse needs across sociodemographic and health-related factors, to improve population health and reduce social gradients.

## Supplementary Information

Below is the link to the electronic supplementary material.

Supplementary Material 1

## Data Availability

The Controller for this public health survey (NIPH) will deposit the data in a publicly available data repository: https://helsedata.no/en/. For more information, contact the first author.

## Abbreviations

ANCOVA: Analysis of covariance
BIC: Bayesian Information Criterion
EQoL-3: Essential QoL-3
NCPHS: Norwegian Counties Public Health Surveys
NIPH: Norwegian Institute of Public Health
PD: Perceived Discrimination
QoL: Quality of Life
LL: Log likelihood
LRX^2^: Likelihood ratio chi-square
SCL-5: Mental distress - a shortened version of the Hopkins Symptom checklist
SES: Socioeconomic Status
