# Supplementary material for: Discrimination experiences and their associations with sociodemographic factors, health and quality of life—a latent class analysis
Source: Health Qual Life Outcomes. 2026 Feb 19;24:42. doi: 10.1186/s12955-026-02502-2 (PMC13020280; doi:10.1186/s12955-026-02502-2)
Supplement: Supplementary file 1 — Supplementary Material 1 [file 12955_2026_2502_MOESM1_ESM.docx]

**Supplemental file:**

**Figure 3 Health and quality of life (QoL) outcomes across the latent classes**

Note: Estimated mean values from unadjusted (ANOVA) analyses for the different perceived discrimination (PD) classes. The blue line represents the QoL score (EQoL-3), the orange line represents self-rated health, and the green line represents mental distress.
